# Pharmacists’ Prescribing in Saudi Arabia: Cross-Sectional Study Describing Current Practices and Future Perspectives

**DOI:** 10.3390/pharmacy8030160

**Published:** 2020-09-02

**Authors:** Alyaa M. Ajabnoor, Richard J. Cooper

**Affiliations:** 1 Pharmacy Practice Department, Faculty of Pharmacy, King Abdulaziz University, P.O. BOX 80324, Jeddah 21589, Saudi Arabia; 2 School of Health and Related Research, University of Sheffield, Sheffield S1 4DA, UK; richard.cooper@sheffield.ac.uk

**Keywords:** Saudi Arabia, pharmacist prescribing, attitude, prescribing model, questionnaire

## Abstract

Pharmacist prescribing is being increasingly undertaken to better use their skills and reduce the workload of existing prescribers such as doctors, often using formal processes to legitimate these activities. In developing countries like Saudi Arabia, however, pharmacists’ prescribing remains informal with no legislation or formal training and there is a lack of research and understanding into such practices. Therefore, we aimed to describe current pharmacist prescribing practices in Saudi Arabia and explore pharmacists’ views about pharmacists’ prescribing. This is a cross-sectional survey study using an online questionnaire of hospital pharmacists in Saudi Arabia about pharmacists’ prescribing, and associated views about prescribing legislation and barriers to implementing pharmacist prescribing. Over a quarter (28.5%) of pharmacists reported themselves as prescribers, 49% were following a collaborative prescribing model, 18% independent prescribing, and 33% were doing both. Ninety percent of prescribers reported confidence in prescribing the appropriate treatment and 92.3% perceived they will benefit from more prescribing training. Healthcare practice culture and pharmacist’s competency were identified as barriers. There is an overall support for pharmacists’ prescribing in Saudi Arabia among this sample of hospital pharmacists, with limitations in resources and the absence of standardized prescribing training being perceived as key barriers to pharmacists’ prescribing.

## 1. Introduction

Over the past few decades, the role of the pharmacist in many countries has evolved from being a compounder and a supplier of medicines, toward a patient-care provider. This involves a range of new responsibilities that includes complex medication management, screening for chronic disease, educating patients, transition of care, drug prescribing, and health promotion activities [1,2,3]. Early involvement of pharmacists in the prescribing process by granting them the right to prescribe can help in optimizing medication use [4]. The practice of non-medical prescribing (NMP) emerged to meet patients’ needs in terms of timely and convenient access to prescribed drugs [5]. As the name suggests, NMP is undertaken by healthcare professionals from non-medical backgrounds such as nurses, pharmacists, and other allied healthcare professionals who, after receiving training in prescribing are granted the legal authority to prescribe medications [6]. The recognition of pharmacists in developed countries as an underutilized healthcare group was a driver to this change in prescriptive authority [5,7]. At the forefront of these NMP changes were the United Kingdom (UK), the United States of America (USA) and Canada, where nurses at first were allowed to perform prescribing activities in both hospital settings and ambulatory care clinics [8]. However, there are different prescribing models practiced by pharmacists internationally and with different degrees of independence [4]. Independent prescribing (IP) models involve a healthcare practitioner being solely responsible for diagnosing and assessing patients’ medical condition and any associated medicine prescribing [9]. Supplementary prescribing (SP) involves a voluntary partnership between an IP (often a doctor) and a supplementary prescriber, using a patient-centered clinical management plan (CMP) agreed by the physician [4]. There is also collaborative prescribing (CP), which is similar to the SP model and practiced mainly in the USA and Canada. CP requires a cooperative relationship between physicians and pharmacists that gives pharmacists the authority to prescribe [4].

Earlier research on pharmacists’ prescribing has explored the perspectives of pharmacists on adopting a prescribing role and how it affects their professional responsibility. A systematic review of stakeholders’ views and experiences of pharmacist prescribing that included 65 studies from limited number of countries reported that the main drivers to pharmacists adopting a prescribing role were better patient management, improving self and professional confidence, developing a clinical role, reducing therapy costs and patient satisfaction [10]. Moreover, many international studies have reported pharmacist prescribing benefits relating to improved patients’ outcomes, reduced physician workload, more accessibility to healthcare services, better utilizations of pharmacist’s skills, and improved job satisfaction [10]. A thematic review of literature relating to SP in the UK reported that nurse and pharmacist practitioners were generally confident about SP and that it has been well implemented in different clinical settings in the UK [11]. The review also found that time, funding, and primary care strategy were significant barriers to the implementation of SP. Evidence from hospital pharmacists in the USA suggested that the absence of support from physicians and other medical staff were key barriers, while support from senior administrators and physicians, along with pharmacists’ training and their willingness to participate in collaborative drug-therapy management were important facilitators [12]. Previous studies have also reported that pharmacists face difficulties in making prescribing decisions, which reflects the limitations in pharmacists training and education that could also represent a barrier to any pharmacist prescribing model [13,14].

In the Kingdom of Saudi Arabia (KSA), pharmacy practice has evolved dramatically over the past 30 years, with currently 28 pharmacy schools granting bachelor’s in pharmacy (BSc.Pharm) or Doctor of Pharmacy (PharmD) degree, and the establishment of postgraduate residency training programs [15]. Although, there is still inconsistency in the names of pharmacy baccalaureate programs or degree names in KSA, such as “Pharmacy”, “Clinical Pharmacy”, “PharmD” and “BSc.Pharm”, with an increase in pharmacy schools offering PharmD degree, all these programs qualify graduates to practice as pharmacists in KSA [16,17]. In the community setting, the role of pharmacists in KSA is limited to dispensing medications and counseling patients, and traditionally in hospitals, pharmacists in KSA have been involved in verifying prescriptions, dispensing, management of stored medications and pharmaceutical supplies [15]. Clinical pharmacy practice in KSA is relatively well established mainly at tertiary care hospitals and is underpinned by pharmaceutical care principles that originated in the US [18]. The majority of clinical pharmacists in KSA have completed postgraduate residency training (i.e., postgraduate year one, PGY1) or specialized training (PGY2) either in national or overseas training programs. On an institutional level, clinical pharmacists in KSA are involved in running clinics for anticoagulation, cardiology and ambulatory care, and practice within a collaborative agreement with physicians that allow pharmacists to prescribe medications and order laboratory tests within the terms and conditions of the agreement. Pharmacists with prescriptive authority are usually experienced clinical pharmacists who have complete postgraduate training (PGY1 and/or PGY2) [15]. However, these activities are currently still considered informal forms of prescribing as there is no national legislation that supports any official prescribing by pharmacists. For this reason, and due to the scarcity of research that addresses the role of pharmacists as prescribers in KSA, this study aimed to provide quantitative evidence of the current practice of pharmacists’ prescribing in KSA and explore their perspectives for further extending the role of pharmacists to prescribers.

## 2. Materials and Methods

### 2.1. Study Design

This study utilized a cross-sectional descriptive design, in which an electronic survey was developed and used to collect quantitative data about Saudi pharmacists’ prescribing activities and explore their attitude on current practice and their willingness to practice as prescribers. The study was approved by the research ethics committee at the School of Health and Related Research, University of Sheffield, UK (application number: 025528).

### 2.2. Sampling

The study started from the 1st of July 2019 and survey dissemination continued for 8 weeks until the 31st of August 2019. The target population was registered hospital pharmacists in KSA; pharmacists working only in retail pharmacies or non-hospital setting were excluded from the study. Licensed pharmacists working in hospital settings of 300 bed capacity or more were invited to participate in this study. The study was conducted at hospitals located in different regions in KSA (e.g., Makkah, Riyadh, Eastern province, Qassim, Madinah). Twenty-six eligible hospitals were identified using the MOH statistics book for the year 2018 [19]. Hospital pharmacists were also approached via two professional societies in KSA. The first was the Saudi Society of Clinical Pharmacy (SSCP), which is a sub-branch of the Saudi Commission for Health Specialties (SCHS) established in 2018, which represents clinical pharmacists in KSA; at the time of conducting this study the society had 153 pharmacist members [20]. The second was the Saudi Oncology Pharmacy Assembly (SOPA), which is part of the Saudi Oncology Society established in 2018 to support and advance oncology pharmacy practice in KSA, and include 60 pharmacist members [20,21]. Sample size calculation was done based on the number of pharmacist members in the SSCP and SOPA. Since the number of employed pharmacists at each of the 26 eligible hospitals was not disclosed to the investigators until pharmacy directors confirmed their participation, it was assumed that on an average 50 pharmacists were employed at each hospital. Using an online sample calculator (Raosoft, Inc, Seattle, Washington, USA; http://www.raosoft.com/samplesize.html), with a chosen accepted error margin of 5%, a 95% confidence level and a 50% response distribution within the described population, the minimum required sample size was 309 participants.

#### 2.2.1. Questionnaire Development

An electronic survey was developed using Google forms to collect data from participants. All questions included in the survey were developed to collect answers in a complete anonymized form to protect anonymity. The survey instrument included 25 questions divided into three sections: the first captured demographic details such as participants’ years of practice, qualifications, practice settings, and workload (Table 1); the second section assessed participant opinions and perspectives on different aspects of pharmacists’ prescribing in KSA; the final section was completed only by pharmacists who self-reported undertaking prescribing duties, to explore prescribing models used, commonly prescribed medications and views on their prescribing experience. Attitudinal questions used a 5-point Likert scale to capture responses to a series of statements. Open-ended questions were included to allow participants to elaborate more on the barriers and facilitators for adopting pharmacists’ prescribing practice in KSA and add in any additional comments they felt were relevant to this topic. All survey items were designed in English, as all pharmacy graduates in KSA have received their undergraduate education in English [16]. The developed survey instrument went through a pilot process to assess the appropriateness of the survey in collecting the required data from pharmacists and the time needed to complete the electronic questionnaire. A pilot stage was performed on a group of five licensed pharmacists in KSA, and they were asked to provide feedback on their experience in completing the survey. Pharmacists who took part in the pilot study were asked not to participate in the main survey study.

#### 2.2.2. Data Collection

Pharmacists were invited to participate in this study via an email that was sent from the SSCP and SOPA. In addition, pharmacy directors at eligible hospitals were contacted by e-mail and asked to participate by completing the electronic survey and disseminating the survey to pharmacists working at their institutions. To mitigate multiple completions of surveys by the same pharmacist, a specific note was added at the start of the electronic survey asking participants to complete the survey only once.

#### 2.2.3. Approach to Analysis

All quantitative data collected from participants were exported from Google forms and imported for analysis to Statistical Package for Social Sciences (SPSS) version 25. Descriptive analysis of percentages and frequencies was undertaken for the categorical variables. Continuous variables were reported as means and standard deviations (SD), while median and interquartile ranges (IQR) were reported for variables with skewed distribution. Inferential statistics were carried out using Chi-square (χ2) test to explore the possible associations between pharmacists’ prescribing status and key independent variables. Independent t-test was also used to determine if there is a difference between the average weekly working hours between participants who prescribe and those who do not. Furthermore, Fisher’s exact test was used to determine if there is a difference between the two groups in variables where the groups were small such as ages, years of practice and monthly income. Correlation analysis was undertaken using Spearman’s rank correlation to investigate the strength of associations between Likert scaled variables (e.g., awareness of legislation or level of confidence of prescribing pharmacists) and ordinal variables (e.g., years of experience and qualifications). All responses were handled in a complete case analysis, and to limit the effect of missing data, all questions included in the survey tool were required to be filled by respondents, except for two questions (i.e., monthly income and the open-ended question on the barriers and facilitators of pharmacists’ prescribing). For all the statistical tests that were undertaken, a *p* value of < 0.05 was considered statistically significant. All responses reported in the open-ended questions were analyzed separately using the thematic approach proposed by Marshall and Rossman [22]. This involved one investigator (AA) reading each open-ended response several times and becoming engaged and familiar with the data and being able to reflect on possible themes discussed by participants. Then, an inductive coding approach was applied where all codes arise directly from participants’ responses. The coding process was performed by AA and completed when no more codes could be identified from the data. Then, the identified descriptive themes were evaluated and compiled for data analysis.

## 3. Results

### 3.1. Study Sample and Response Rate

The survey was disseminated to 153 pharmacist members in SSCP, and 60 pharmacist members in SOPA. Out of the 26 eligible institutions contacted and invited to participate in this survey study, only 14 hospitals agreed to participate with a total of 712 pharmacists working at these institutions who received an invitation to complete the electronic survey. In total, 141 responses were received, with 4 being excluded as they were from respondents from retail pharmacies and non-hospital setting giving a total of 137 responses and a response rate of 14.8%.

### 3.2. Respondents Characteristics

Table 1 presents a summary of participant demographic characteristics. Of the 137 pharmacists who completed the survey just over half (52.5%, *n* = 72) were females. Around half of the respondents had less than five years of professional experience (47.5%, *n* = 65) with nearly a quarter reporting 6 to 10 years of experience (24%, *n* = 33). Forty percent of participants (*n* = 55) held a Pharm.D degree as a qualification to practice pharmacy and 19.7% (*n* = 27) had a BSc.Pharm degree. Twenty-three percent of respondents (*n* = 32) completed pharmacy residency training of which 13.1% (*n* = 18) were graduates of PGY1 training programs, and 10.2% (*n* = 14) completed PGY2 specialized training programs. The majority of respondents were working at governmental hospitals (76.6%, *n* = 105), and the majority were working at Makkah region (57%, *n* = 78) followed by Riyadh region (19%, *n* = 26). Just over one third of the respondents were working at clinical pharmacy settings (35.9%, *n* = 49) who are practicing as pharmacy practice resident or as clinical pharmacist or as specialized clinical pharmacist. This was followed by respondents working at an outpatient pharmacy setting (24%, *n* = 33).

### 3.3. Perspectives on Pharmacists’ Prescribing

Just under half of the respondents (47.5%, *n* = 65) were aware that there is no national legislation to support pharmacist prescribing in KSA (Table 2). The majority of respondents (77%, *n* = 105) agreed that there was a need for legislation to support pharmacists’ prescribing in KSA, which was not correlated with age or experience (r = 0.177, *p* = 0.282; r = 0.048, *p* = 0.770, respectively) but was for highest professional/academic qualifications (r = 0.211, *p* = 0.014).

However, opinions were more divided regarding the prescribing model favored: (41.6% *n* = 57) agreed that pharmacists should prescribe independently of physicians but more (69.3%, *n* = 95) agreed with a collaborative prescribing model. The majority also felt that prescribing should be limited to competent clinical pharmacists (65%, *n* = 89) and (90.5%, *n* = 124) agreed that pharmacists should receive training specific to the therapeutic areas they will prescribe within (Table 2). There was a positive correlation between respondents’ attitude toward pharmacists’ prescribing training and highest professional/academic qualifications (r = 0.264, *p* = 0.002). There was a general agreement with all the statements that described the potential benefits of implementing pharmacist prescribing. These included increasing patients’ access to medications (82.5%, *n* = 113) and improving the overall quality of care for patients (76.6%, *n* = 105). Participants expressed less agreement with the benefit of reducing prescribing errors (63.5%, *n* = 87), and with patients avoiding physician follow-up (51.8%, *n* = 71) (Table 2).

In relation to the impact that prescribing could have for pharmacists, most respondents (89.1%, *n* = 122) agreed that prescribing will increase pharmacist’s professional responsibility. The majority (84.7%, *n* = 116) agreed that prescribing allows utilization of pharmacist’s skills and experience and offered more job satisfaction (81%, *n* = 111). However, 70.1% (*n* = 96) of respondents acknowledged that prescribing would increase pharmacist’s workload (Table 2).

### 3.4. Perceived Barriers to Pharmacist Prescribing

Several key themes emerged from participants’ responses, reflecting views about the need for legislation, concerns about pharmacist training and competency, support from doctors, existing healthcare practice cultures, and sufficient resources to fund pharmacist prescribing. These are now considered in turn:

(a) **Legislation**—Lack of prescribing legislation for pharmacists and specifically at a national level was a frequently cited barrier that was perceived to prevent pharmacist prescribing in KSA (Box 1).
  Box 1 **Legislation.**
  “There is NO national legislation that supports pharmacists prescribing”
  “Lack of national legislations to back up and protect pharmacists”
  “Lack of cooperation between government and institutions to make sure that pharmacists are well oriented to prescribe”
(b) **Pharmacists training and competency**—participants highlighted the issue of lack of appropriate training that would enable pharmacists to prescribe, both in terms of competency but also as a qualification. There was an emphasis on the need for specified training programs in therapeutic areas along with national certification before granting pharmacists prescriptive authorities (Box 2).
  Box 2 **Pharmacists training and competency.**
  “I think the one important thing to achieve that level is to have qualified pharmacists who have enough experience to minimize the risk of prescribing mistakes”
  “[…] need specified training, in each specialty…. and need condensed courses and programs to improve pharmacist’s knowledge and practice”
  “Still need time and more practice”
  “Need appropriate training and national competency certification”
(c) **Physicians’ perceived negativity**—most participants believed that there was significant resistance from physicians, which represented a key barrier to implementing any form of pharmacist prescribing practice in KSA (Box 3). This appeared to be related to aspects such as physicians’ lack of awareness of pharmacists and not wanting to work in an inter-disciplinary way.
  Box 3 **Physicians’ perceived negativity.**
  “Lack of physicians’ support and collaboration”
  “Physicians are not aware of the extent of pharmacists knowledge and abilities”
  “Doctors don’t like pharmacist to intervene in their job”
  “This idea is not accepted by multidisciplinary teams”
  “Physicians resistance only, as patients do trust pharmacists and ask them for advice about their medical conditions”
(d) **Healthcare practice culture**—many participants believed that there were certain norms within healthcare practice in KSA that represents a barrier to extending pharmacist role to a prescriber. Many respondents referred to how patients are used to seeking medical care only from physicians and that patients view pharmacists only as a dispenser of medications who are not involved in patients’ care (Box 4).
  Box 4 **Healthcare practice culture.**
  “The patient trusts the physician more than pharmacist”
  “Expectations from patients and other healthcare providers”
  “For many years prescribing medications was limited to medical doctors”
  “The nature of how things are processed in the hospital…each person has a specific role”
(e) **Limited resources**—participants also suggested that there are limitations in resources that could facilitate the adoption of pharmacists’ prescribing in KSA. In this context, participants mentioned that pharmacists do not have enough time to practice as prescribers giving their workload and the demanding nature of their traditional roles as pharmacists. In addition, they mentioned that pharmacists do not have full access to patient’s information to allow them to practice as prescribers (Box 5).
  Box 5 **Limited resources.**
  “No enough time to practice as prescribers”
  “Pharmacists workload”
  “The pharmacists are not allowed to get full information about patient case and are not trusted by patients or physicians”

### 3.5. Pharmacist Prescribing Practice

Just over a quarter of pharmacists (28.5%, *n* = 39) described themselves as prescribers (Table 3). From this subgroup of prescribing respondents just under half (48.7%, *n* = 19) described their prescribing as collaborative, in which they initiated and monitored medicines according to a CMP that has been agreed in conjunction with a physician. In contrast, only 18% (*n* = 7) followed an independent prescribing model, in which they are responsible for assessing patients and making decisions about their CMP including prescribing medications. The remaining third (33.3%, *n* = 13) reported prescribing using both independent and collaborative prescribing models. When asked about the source of their prescribing authority, around half of prescribing pharmacists (51.3%, *n* = 20) cited both a collaborative agreement with a medical team as well as approval by their institution as being needed to prescribe; around a third (30.7%, *n* = 12) reported a collaborative agreement with a medical team only, and the remaining 18% (*n* = 7) of pharmacists were granted the authority to prescribe by their institution only (Table 3). More than half of prescribing pharmacists (53.8%, *n* = 21) have received training in prescribing and the majority had access to patients’ medical records during prescribing (82%, *n* = 32). The median number of prescriptions that were prescribed by pharmacists in a typical week was reported to be 10 (IQR = 5–35), and it involved spending a median of 15 min to complete a prescription (IQR = 5–20) (Table 3).

**Figure 1:**
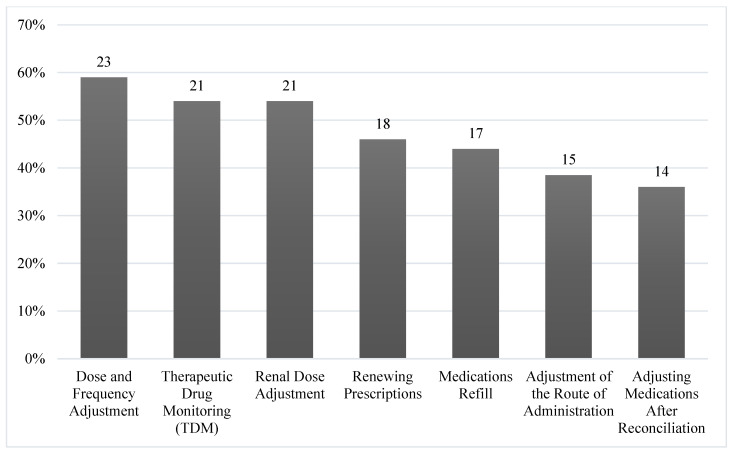
Domains of prescribing practice (*n* = 39).

**Figure 2:**
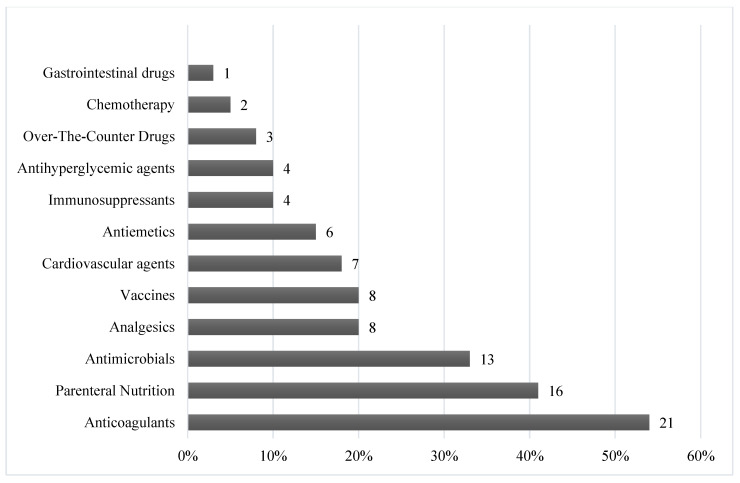
Common prescribed agents by therapeutic class (*n* = 39).

The most reported prescribing activities (59%, *n* = 23) involved dose and frequency adjustments, followed by renal dose adjustments and therapeutic drug monitoring (TDM) (53.8%, *n* = 21) (Figure 1). The most prescribed medications by respondents (Figure 2) were anticoagulants (53.8%, *n* = 21), followed by parenteral nutrition and antimicrobials reported by (41%, *n* = 16) and (33.3%, *n* = 13) of participants, respectively.

### 3.6. Experience of Prescribing Pharmacists

Table 4 summaries participants’ (*n* = 39) responses to statements on their prescribing experience. Most respondents were confident to prescribe in their area of practice (89.7%, *n* = 35) and believed that prescribing makes their job more satisfying (87.2%, *n* = 34). Results showed a positive correlation between the age of respondents and their level of confidence to prescribe (r = 0.329, *p* = 0.041). No such correlation was identified with respondent’s experience or their highest professional/academic qualifications (r = 0.205, *p* = 0.212; r = 0.142, *p* = 0.389, respectively). Respondents expressed awareness of their limitations as prescribers (92.3%, *n* = 36) and perceived that more training would benefit them as prescribers (92.3%, *n* = 36). Most of the participants agreed that prescribing increased their workload (74.4%, *n* = 29), and they also expressed satisfaction at the level of training they completed before prescribing (74.4%, *n* = 29), and support received from the medical teams (76.9%, *n* = 30) or the institution (66.7%, *n* = 26). Participants were asked about their preferences for prescribing practice and 38.6% (*n* = 15) agreed that both collaborative agreement with physicians and also independent prescribing in their area of expertise were preferable; a further 38.6% (*n* = 15) agreed with collaborative prescribing only and were either neutral or negative about independent prescribing, and only 17.9% (*n* = 7) agreed with independent prescribing but were neutral or negative about collaborative practice; 5.1% (*n* = 2) were neutral about both practices. Additionally, (35.9%, *n* = 14) of participants faced resistance from physicians or other healthcare professionals during their prescribing practice. A negative correlation was identified between the resistance participants have faced from physicians or other healthcare providers during their prescribing practice and their highest professional/academic qualifications (r = −0.393, *p* = 0.013). No correlation was identified with respondent’s experience or their age (r = 0.155, *p* = 0.347; r = −0.167, *p* = 0.310).

### 3.7. Comparison between Prescribing and Non-prescribing Pharmacists

Prescribing and non-prescribing pharmacists were compared in relation to key demographics. (Table 5). Prescribers were more likely to have a Pharm.D degree (*p* = 0.003), completed residency training (*p* = 0.001), and practice in clinical rather than a non-clinical pharmacy setting (*p* = 0.001). However, there was no significant difference identified in the gender between the two groups (*p* = 0.849) or the type of healthcare institutions participants work in (*p* = 0.663). Additionally, both prescribing and non-prescribing pharmacists were from similar age groups (*p* = 0.305), had similar years of practice (*p* = 0.466), monthly income (*p* = 0.269), and average working hours in a typical week (*p* = 0.141).

## 4. Discussion

This study aimed to describe the informal pharmacist prescribing practices in Saudi Arabia, and the perceptions of hospital pharmacists on extending the role of pharmacists to that of prescribers. Results revealed that hospital pharmacists hold a positive attitude toward introducing legislation to support pharmacist prescribing formally and nationally in KSA and feel that prescribing would better utilize pharmacist’s skills and experience. Just over a quarter of participants identified themselves as current prescribing pharmacists and were mainly practicing in clinical pharmacy settings with collaborative practice agreements with physicians. Of note was that around half of prescribing pharmacists were practicing as independent prescribers. However, participants identified limited resources and healthcare practice culture as commonly perceived barriers to formalize this practice and introduce legislation to support it.

Awareness was divided over the current lack of national legislation to support pharmacists’ prescribing in KSA. Although, pharmacy schools in KSA provide courses for pharmacy practice regulations as part of the curriculum [23], these findings reflect the limitations in pharmacist’s awareness of the MOH health practice regulations. Furthermore, participants expressed more agreement with limiting pharmacists’ prescribing to competent clinical pharmacists (65%), rather than allowing pharmacists, in general, to prescribe (38%). This may be related to clinical pharmacists in KSA having completed residency training programs that equipped them with advanced clinical skills and knowledge, allowing them to serve as direct patient-care providers [24,25]. In light of this, in the recent review by Al-Omi et al., pharmacist prescribing was declared as a new initiative in KSA, and as a new project it requires special training and education including clinical pharmacists, dispensing pharmacists, and technicians as well [26].

Results also showed that more than half of participants believed that pharmacists’ prescribing improved the quality of care for patients, reduced prescribing errors, and helped patients avoid physician’s follow-up. According to earlier studies that evaluated the outcomes of pharmacist’s medication therapy management compared to traditional medical care, when pharmacists managed drug-therapy initiation and monitoring, this resulted in patient outcomes equal and sometimes superior to those of standard care [12,27]. Additionally, pharmacists’ prescribing was perceived to reduce doctors’ workload in this study, which has also been reported in previous research involving the positive views of policymakers [28,29,30], doctors [31], and pharmacists [32]. This has benefits in terms of physicians having more time to deal with more complex cases, leaving more routine or pre-diagnosed patients to the care of prescribing pharmacist.

More negatively, participants identified limitations in existing training and national certification programs and felt that these were not appropriate to allow pharmacists to become prescribers; physicians were also not felt to be supportive of pharmacists’ prescribing as they are unaware of pharmacist’s abilities and knowledge. Moreover, respondents perceived that the demanding nature of the pharmacist profession to represent a barrier for pharmacists to adopt a prescribing role. These finding are similar to previous research in which lack of time for pharmacists to take additional workload was identified as a barrier for pharmacist prescribing [33,34,35,36], along with limited support from physicians [31,37,38]. Similar negativity emerged in some aspects of the findings by Abdel-Latif [38], who sampled doctors in Saudi Arabia and identified a lack of awareness and willingness to accept advice on prescription changes.

In relation to current self-reported prescribing practice and models, this study suggests that collaborative prescribing is the most common, either with local authority or combined with independent prescribing. Those reporting only independent prescribing reflected 18% of prescribers. Evidence in the literature makes comparisons difficult and relatively little research have quantified the proportion of different NMP, which may be related to definitional issues and also different settings and practitioners [39]. One study in the US used an analysis of state legislation and identified a continuum and noted that categories of pharmacy NMP were not mutually exclusive and that collaborative prescribing guidelines were more common [40]. Given the infancy of NMP in KSA, the identification of more collaborative prescribing may reflect findings in other research where such prescribing—for example supplementary prescribing—might be more suited to those without previous experience as a “stepping stone” [41].

Pharmacists in this study were more commonly involved in prescribing for anticoagulants, parenteral nutrition, and infectious diseases or antimicrobials, which included prescribing activities like TDM and dose adjustments. The scope of this prescribing practice is similar to that in the USA, where most hospitals with established collaborative prescribing by pharmacists, allowed pharmacists to adjust drug strengths, order lab tests, and modify drug’s frequency for treatment areas similar to the ones in our study findings [12]. Moreover, in the USA within hospital settings pharmacists are authorized to adjust heparin infusions, and provide outpatient pain management, including prescribing of supplementary therapy such as antihistamines, laxatives, benzodiazepines and antiemetics [42]. In primary care settings in the UK, pharmacists with supplementary prescribing authority where mainly involved in clinical areas like hypertension, coronary heart disease, and diabetes. As for secondary care settings, TPN was identified as the specialty with the highest number of supplementary prescribers, and more pharmacists are being trained to prescribe in areas such as HIV, cystic fibrosis, and surgery/orthopedics [43].

Confidence in prescribing was positively correlated with pharmacists’ age, which may be related to the amount of experience gained by older pharmacists over time, and that increased confidence comes with increased age. Results also identified a negative correlation between physicians’ perceived resistance to pharmacists’ prescribing and the highest qualification for the prescribing pharmacist, indicating less resistance from physicians to prescribing pharmacists who are holding higher qualifications. Earlier research had highlighted the confidence factor in pharmacists’ prescribing practice. It was reported that confidence in prescribing comes from a defined area of competence [44], and that non-medical prescribers are cautious when prescribing but their confidence improves with good support from physicians [45,46]. Moreover, the majority of respondents (87%) agreed that prescribing did make their job more satisfying. This is also consistent with evidence from previous studies exploring pharmacists’ views on the impact of pharmacists’ prescribing, in which many pharmacists believed that prescribing would increase their job satisfaction [36,44,45,46,47].

### 4.1. Strengths and Limitations

This is the first study to explore NMP attitudes and practices in KSA and using a quantitative survey design has revealed unique insights into pharmacists’ beliefs and current prescribing practice. Limitations in the study relate to the use of a self-report questionnaire, and respondents could have been self-selected with a greater interest in the study topic and, therefore, pharmacist’s prescribing results might not be specifically reflective of other—particularly non-prescribing pharmacists. The overall response rate to this survey was 14.8%, which is lower than some other studies using surveys with pharmacists [32,35]. Although participants were told not to complete the survey more than once, some of the pharmacists invited to participate from the 14 hospitals institutions could also have been invited as members of the professional organizations, caution is needed in generalizing from these findings to all pharmacists in KSA in the hospital setting. Additionally, assessing for non-response bias was not possible since information on pharmacists who did not respond was not available to the investigator in order to assess the likelihood of non-response bias by comparing the characteristics of responders and non-responders.

### 4.2. Implications for Policy, Practice and Future Research

In articulating hospital pharmacists’ views and current local and informal practices, this study adds weight to claims that pharmacist’s prescribing in KSA should be legitimated through the introduction of national legislation. An implication of this is that hospitals should then adopt national prescribing arrangements rather than institutional ones. A further implication is that national legislation would standardize requirements needed for pharmacists to undertake prescribing. This could be in the form of pharmacist’s completion of prescribing training similar to the training requirements that is being implemented in developed countries, in which pharmacists are required to achieve a certain level of prescribing competency and pass tests to enable them to act as prescribers [48,49,50]. Finally, research is required to explore the views and opinions of stakeholders including not only pharmacists but also physicians and other healthcare professionals who are involved in patient care and prescribing to reflect their views on pharmacist’s prescribing, and also to evaluate the impact of pharmacists’ prescribing, on the quality of patient care, healthcare costs, and patient’s satisfaction.

## 5. Conclusions

There is an overall support for pharmacist prescribing in Saudi Arabia among this sample of hospital pharmacists. There is a general agreement by pharmacists for the need of specific prescribing training before allowing pharmacists to prescribe and national legislation to legitimize and standardize practice. Just over a quarter of respondent pharmacists were practicing as prescribers. Informal pharmacists’ prescribing activities were identified mainly within a collaborative practice agreement with physicians and practiced mainly by clinical pharmacists. Most of prescribing pharmacists were authorized to perform dose and frequency adjustments and TDM, and they were most frequently involved in prescribing activities for anticoagulants, parenteral nutrition, and antimicrobials. A key demographic difference between prescribing and non-prescribing pharmacists in KSA was the completion of residency training and practice in a clinical pharmacy setting. In general, there is support from tertiary care hospitals to pharmacists’ prescribing, and collaborative practice agreements are approved by hospital administrations in most cases. Healthcare practice culture and limitations in the availability of standardized prescribing training are key barriers to the legislation of pharmacists’ prescribing in Saudi Arabia.

## Figures and Tables

**Table 1:** Pharmacists’ respondent characteristics (*n* = 137).

Demographic Variables	% (*n*)	
Females	52.5 (72)	
Age group	20–29 years	43.8 (60)
	30–39 years	39.4 (54)
	40–49 years	13.1 (18)
	>50 years	3.7 (5)
Practice setting	Government hospital	76.6 (105)
	Private hospital	23.4 (32)
Geographical region	Makkah	57 (78)
	Riyadh	19 (26)
	Eastern Province	16.8 (23)
	Madinah	2.9 (4)
	Hai’l	1.5 (2)
	Qassim	0.7 (1)
	Tabuk	0.7 (1)
	Jizan	0.7 (1)
	Baha	0.7 (1)
Pharmacy practice area	Administration	13.1 (18)
	Inpatient pharmacy	16.8 (23)
	Outpatient pharmacy	24 (33)
	Clinical pharmacy	35.9 (49)
	Others	10.2 (14)
Years of practice as a qualified pharmacist	<5 years	47.5 (65)
	6 to 10 years	24 (33)
	11 to 15 years	10.2 (14)
	16 to 20 years	11.7 (16)
	21 to 25 years	4.4 (6)
	>25 years	2.2 (3)
Highest professional or academic degrees	BSc.Pharm	19.7 (27)
	Pharm.D	40.2 (55)
	MPharm	2.2 (3)
	PG Diploma	4.4 (6)
	Master’s degree	8 (11)
	PGY1	13.1 (18)
	PGY2	10.2 (14)
	Ph.D	2.2 (3)
Professional positions	Outpatient pharmacist	24 (33)
	Inpatient pharmacist	19.7 (27)
	Pharmacy practice resident	8 (11)
	Clinical pharmacist	10.2 (14)
	Specialized clinical pharmacist	16.1 (22)
	Deputy director	1.5 (2)
	Pharmacy director	8.8 (12)
	Other	11.7 (16)
Monthly income ^$^	<SR * 10,000	12.4 (17)
	SR 10,000–SR 20,000	66.4 (91)
	SR 20,000–SR 30,000	9.5 (13)
	SR 30,000–SR 40,000	5.8 (8)
	>SR 40,000	2.9 (4)

^$^ Responses may not add to 100% because of 4 missing responses. * SR: Saudi Riyal.

**Table 2:** Respondents’ opinions on statements regarding pharmacists’ prescribing (*n* = 137).

Statements	Responses* % (*n*)					
	Strongly Agree	Agree	Neutral	Disagree	Strongly Disagree	Median Score (IQR) *
There is national legislation that support pharmacists’ prescribing in KSA	5.8 (8)	24.8 (34)	21.9 (30)	30.7 (42)	16.8 (23)	3 (2–4)
Legislation to support pharmacist prescribing should be available in KSA	35.1 (48)	41.6 (57)	14.6 (20)	5.8 (8)	2.9 (4)	4 (4–5)
Pharmacists should be allowed to prescribe independently of the medical team	14.6 (20)	27 (37)	23.4 (32)	24 (33)	11 (15)	3 (2–4)
Pharmacists should only be allowed to prescribe in collaboration with physicians	28.5 (39)	40.8 (56)	19 (26)	8.8 (12)	2.9 (4)	4 (3–5)
Prescribing should be limited to competent clinical pharmacists	32.1 (44)	32.8 (45)	16.1 (22)	11 (15)	8 (11)	4 (3–5)
Pharmacists should be trained in specific therapeutic areas before they are allowed to prescribe	61.3 (84)	29.2 (40)	5.1 (7)	2.9 (4)	1.5 (2)	5 (4–5)
Pharmacist prescribing will allow greater patient access to medications	40.2 (55)	42.3 (58)	14.6 (20)	2.2 (3)	0.7 (1)	4 (4–5)
Pharmacist prescribing helps patients avoid physician follow-up	21.2 (29)	30.7 (42)	27.7 (38)	12.4 (17)	8 (11)	4 (3–4)
Pharmacists’ prescribing reduces prescribing errors	33.6 (46)	29.9 (41)	28.5 (39)	5.8 (8)	2.2 (3)	4 (3–5)
Pharmacists’ prescribing will increase the quality of care for patients	38.7 (53)	37.9 (52)	19.7 (27)	2.2 (3)	1.5 (2)	4 (4–5)
Pharmacist prescribing increases pharmacists professional responsibility	49.6 (68)	39.4 (54)	7.3 (10)	2.2 (3)	1.5 (2)	5 (4–5)
Prescribing increases pharmacist’s workload	29.2 (40)	40.9 (56)	22.6 (31)	5.8 (8)	1.5 (2)	4 (3–5)
Prescribing increases pharmacist’s job satisfaction	37.9 (52)	43.1 (59)	11.7 (16)	5.1 (7)	2.2 (3)	4 (4–5)
Pharmacists’ prescribing allow greater utilization of pharmacist’s skills and experience	46.7 (64)	37.9 (52)	11 (15)	2.9 (4)	1.5 (2)	4 (4–5)

* 1 = ’’Strongly disagree’’, 5 = ’’Strongly agree’’.

**Table 3:** Responses from prescribing pharmacists (*n* = 39).

Questions		% (*n*)
Type of prescribing model practiced by the pharmacist	Independent prescribing	18 (7)
	Collaborative prescribing	48.7 (19)
	Both independent and collaborative prescribing	33.3 (13)
Prescriptive authority was given to the pharmacist by…	The institution he/she work in	18 (7)
	A collaborative agreement with the medical team	30.7 (12)
	A collaborative agreement with a medical team that was approved by the administration of the institution	51.3 (20)
Prescribing training received (other than postgraduate clinical training or qualification)	Yes	53.8 (21)
	No	46.2 (18)
Access to patients’ medical records during prescribing	Yes	82 (32)
	No	18 (7)
As a result of pharmacists’ prescribing, doctors are prescribing …	less	41 (16)
	more	35.9 (14)
	The same amount	23.1 (9)
Time spent (in minutes) to complete a prescription including documentation in patients’ records		15 (5–20) *
Prescriptions issued by pharmacists per week		10 (5–35) *

* Median (IQR).

**Table 4:** The opinion of pharmacists on their prescribing experience (*n* = 39).

Statements	Responses *, % (*n*)					
	Strongly Agree	Agree	Neutral	Disagree	Strongly Disagree	Median Score (IQR) *
I am confident to prescribe the appropriate treatment for patients in my practice area	51.2 (20)	38.5 (15)	5.1 (2)	2.6 (1)	2.6 (1)	5 (4–5)
Being a prescriber makes my job more satisfying	46.2 (18)	41 (16)	10.2 (4)	2.6 (1)	0	4 (4–5)
I am aware of my limitations as a prescriber	53.8 (21)	38.5 (15)	7.7 (3)	0	0	5 (4–5)
Prescribing has increased my workload	35.9 (14)	38.5 (15)	12.8 (5)	5.1 (2)	7.7 (3)	4 (3–5)
I am satisfied by the level of training I received before prescribing	41 (16)	33.3 (13)	12.8 (5)	7.7 (3)	5.1 (2)	4 (3–5)
The medical team I work with are cooperative and supportive to my prescribing practice	48.7 (19)	28.2 (11)	12.8 (5)	7.7 (3)	2.6 (1)	4 (4–5)
I faced resistance from physicians or other healthcare professionals during my prescribing practice	12.8 (5)	23.1 (9)	28.2 (11)	23.1 (9)	12.8 (5)	3 (2–4)
My institution had been supportive of pharmacist prescribing	30.8 (12)	35.9 (14)	25.6 (10)	5.1 (2)	2.6 (1)	4 (3–5)
I prefer to prescribe only within a collaborative agreement with physicians	38.5 (15)	38.5 (15)	15.3 (6)	7.7 (3)	0	4 (4–5)
I prefer to practice as an independent prescriber in my area of expertise	25.6 (10)	30.8 (12)	25.6 (10)	10.3 (4)	7.7 (3)	4 (3–5)
Receiving more training in prescribing will benefit me as a prescriber	69.2 (27)	23.1 (9)	7.7 (3)	0	0	4 (4–5)

* 1 = ’’Strongly disagree’’, 5 = ’’Strongly agree’’.

**Table 5:** Comparison between the characteristics of prescribing and non-prescribing pharmacists.

Characteristics	Prescribing Pharmacists (*n* = 39) % (*n*)	Non-Prescribing Pharmacists (*n* = 98) % (*n*)	*p*-Value
Gender			
Male	46.2 (18)	48 (47)	0.849
Female	53.8 (21)	52 (51)	
Age group			
20–29 years	35.9 (14)	46.9 (46)	0.583
30–39 years	46.2 (18)	36.7 (36)	
40–49 years	12.8 (5)	13.3 (13)	
>50 years	5.1 (2)	3.1 (3)	
Doctor of pharmacy degree			
Yes	84.6 (33)	56.1 (55)	0.003
No	15.4 (6)	43.9 (43)	
Complete pharmacy residency			
Yes	59 (23)	9.3 (9)	0.001
No	41 (16)	90.7 (88)	
Healthcare institution			
Governmental	79.5 (31)	75.5 (74)	0.663
Private	20.5 (8)	24.5 (24)	
Practice setting			
Clinical pharmacy	61.5 (24)	25.5 (25)	0.001
Non-clinical	38.5 (15)	74.5 (73)	
Years of practice			
<5 years	41 (16)	50 (49)	0.466
6 to 10 years	31 (12)	21.4 (21)	
11 to 15 years	10 (4)	10.2 (10)	
16 to 20 years	8 (3)	13.3 (13)	
21 to 25 years	5 (2)	4.1 (4)	
>25 years	5 (2)	1 (1)	
Monthly income ^$^			
<SR 10,000	15.4 (6)	11.2 (11)	0.269
SR 10,000–SR 20,000	67 (26)	66.3 (65)	
SR 20,000–SR 30,000	5.1 (2)	11.2 (11)	
SR 30,000–SR 40,000	3 (1)	7.1 (7)	
>SR 40,000	8 (3)	1 (1)	
Average working hours in a typical week	47.3 (23) *	44 (6.2)	0.141

^$^ Responses may not add to 100% because of 4 missing responses. * Mean (SD).
